# Factors associated with meeting homosexual partners at fixed offline locations among MSM recruited through the internet: A cross-sectional survey

**DOI:** 10.1371/journal.pone.0325273

**Published:** 2025-05-28

**Authors:** Weiyong Chen, Zhongrong Yang, Xing Wang, Weiwei Wang

**Affiliations:** 1 Department of HIV/STD Control and Prevention, Zhejiang Provincial Center for Disease Control and Prevention, Hangzhou, Zhejiang Province, China; 2 Science Research Education and Quality Management, Huzhou Center for Disease Control and Prevention, Huzhou, Zhejiang Province, China; 3 Department of Neurosis and Psychosomatic Diseases, Huzhou Third Municipal Hospital, The Affiliated Hospital of Huzhou University, Huzhou, Zhejiang Province, China; 4 Department of Drug Clinical Trial Institution, Huzhou Central Hospital, Affiliated Central Hospital of Huzhou University, Huzhou, Zhejiang Province, China; Jiangsu provincial Center for Disease Control and Prevention, CHINA

## Abstract

**Objective:**

This study aimed to investigate the demographic characteristics of Men who have Sex with Men (MSM) recruited online and identify the factorsassociated with meeting homosexual partners at fixed offline locations.

**Methods:**

Univariate and multivariate logistic regression analyses were used to examine the factors that influenced their meeting up with homosexual partners at fixed offline locations.

**Results:**

A total of 604 MSM were included, with 133 participants (22.02%) meeting homosexual partners at fixed offline locations. Multivariate logistic regression analysis showed that participantswho were willing to engage in commercial sex, engage in behaviors such as alcohol consumption, drug use, or aphrodisiac use during homosexual activities, and hadused HIVPre-Exposure Prophylaxis (PrEP) in the last six months were more likely to meet homosexual partners at fixed offline locations.

**Conclusions:**

The proportion of participants meeting homosexual partners at fixed offline locations was relatively high, emphasizing the need to increase education and awareness among MSM to reduce relatedbehaviors during homosexual activities. Further promotion of the proper use of PrEP and avoidance of commercial sex are essential for lowering the risk of HIV infection among this population.

## 1. Introduction

Acquired ImmunoDeficiency Syndrome (AIDS), caused by the Human Immunodeficiency Virus (HIV), is a severe infectious disease that has emerged as a major global public health concern [1,2]. The HIVepidemic remains particularly serious in certain developing countries and regions, and significantlyimpacts local socioeconomic development and public health [3,4]. Despite advancements in AIDS prevention and control, including the widespread availability of antiretroviral therapy, increased HIV testing coverage, and heightened promotion of AIDS awareness and condom use, challenges persist [5]. For example, Men who have Sex with Men (MSM) have a high infection rate, and conventionalhealth education is difficult to effectively prevent the spread of HIV.HIV infected individuals still face restrictions on their rights to medical treatment and employment, and social discrimination has led to some HIV infected individuals avoiding testing and treatment.The health-related quality of life of individuals infected with HIV is notably compromised [6]. High riskof HIV infection persistsin specific populations, such as MSM, people who use drugs or people in sex work [7]. Public awareness and attitudes towards AIDS need to be furtherimproved, along with strengthening of the monitoring and control systems for AIDS. HIVprevention and control represent an enduring commitment in the realm of public health, necessitating collaborative efforts from governmental bodies, civil society, and individuals to implement comprehensive and targeted measures to achieve sustainable epidemic prevention and control objectives.

Globally, MSM havea significantly elevated risk of infection compared to the general populationbecauseof their specific sexual behaviors, primarily involving anal intercourse [8,9]. The HIVepidemic is notably more severe among MSM in certain countries and regions [10]. Traditional discrimination, stigmatization, and lack of attention and support towards sexual minorities pose obstacles for MSM seeking health services and information [11,12]. In more open and inclusive social environments, MSM enjoy enhanced accessibility to diverse health services and information resources, thereby improving their health outcomes and reducing the risk of HIV transmission. The rapid evolution of the internet as a novel platform for information retrieval and social engagement offers this group a broader avenue for forging social connections and relationships [13].

It has become a common technique to recruit MSM for relevant research through the internet, of particular interest are the circumstances and factors surrounding the meetings of homosexual partners at specific offline venues [14,15]. However, there are few studies on the dynamics and factors affecting MSMrecruited through online recruitmentin their meetings with homosexual partners at fixed offline locations (such as bars, karaoke television, saunas, and parks, etc). This study aimed to address this gap by investigating the underlying factors influencing the social behavior among MSM, aiming for a comprehensive understanding of their social interaction patterns and sexual behavioral traits. This studysought to uncover the scenarios and potential factors associated with MSM meeting homosexual partners at fixed offline locations through online channels. This initiative may enrich sexual health education strategies and advance the sexual health and societal integration of MSM. A thorough understanding of these dynamics and determinants may providenovel insights and research pathways in the realm of sexual health studies, offering valuable references for future research and interventions.

## 2. Materials and methods

### 2.1 Study design and ethical declaration

This cross-sectional survey study was conducted by a non-governmental organization named Ma’anqianxun health information consultation center in Zhejiang province in May 2024 by recruiting MSM online and administering a questionnaire [16]. The research protocol was approved by the Ethics Committee of the Huzhou Center for Disease Control and Prevention (Approval no.: HZ2023003), and all participants provided written informed consent before completing the questionnaire.

### 2.2 Study participants

The study participants were MSM who were recruited via the internet. A total of 626 MSM completed the survey online via WeChat, with 604 providing written informed consent and completing the survey, and 22 declining to participate.

#### 2.2.1 Inclusion criteria.

(1) Being a MSM, (2) age ≥18 years, (3) online recruitment, and (4) provided written informed consent to participate in the survey.

#### 2.2.2 . Exclusion criteria.

(1) Age < 18 years, (2) offline recruitment or at a fixed location, (3) unwillingness to provide informed consent, (4) self-reported mental or cognitive impairments.

### 2.3 Survey content

Our questionnaire design was mainly based on the Chinese MSM sentinel monitoring questionnaire and the college student survey questionnaire [17–19]. After conducting a pre survey and improving the questionnaire variable settings, we conducted a formal questionnaire survey. The survey questionnaire [16] comprised general demographic characteristics (such asage, education level, and average monthly income), sexual behavior-related features (including sexual attitudes, condom usage during sexual activities, having a consistent sexual partner, and recent sexual activity within the past 6 months), receipt of HIV/AIDS prevention services in the previous year (including promotional materials and distribution of condoms, counseling services, and training sessions), HIV testing within the last year, and details regarding the utilization of HIV Pre-Exposure Prophylaxis(PrEP) in the past six months.

### 2.4 Definitions of the related indicators

Commercial sexual behavior refers to sexual activities involving monetary transactions, including sugar-daddy arrangements, prostitution, and solicitation. The receipt of prevention services refers to whether a participant received HIV/AIDS-related preventive services in the previous year, including the distribution of informational materials and condoms, counseling services, and training workshops. A one-night stand was defined as a casual sexual encounter between MSM.

### 2.5 Quality control

The questionnaire was set up on the Wenjuanxing platform, allowing each WeChat ID to complete it once. Effective quality control measures were implemented through questionnaire restrictions, including mandatory questions, logic jumps, and response range limitations to obtain valid questionnaires that met the recruitment criteria. The survey staff received standardized training for using a uniform questionnaire for data collection. Before the survey, the investigators explained the purpose, significance, methodology, and privacy protection policy to the participants; this information was included at the beginning of the questionnaire, and by clicking a checkbox or a button indicating voluntary participation and consent. Participants were informed that the survey aimed to develop prevention strategiesfor HIV and sexually transmitted infectionsamong MSM, that the survey would be anonymous, and that only group data, not personal data, would be analyzed.

### 2.6 Statistical analysis

Statistical analyses were performed using R version 4.4.1.Descriptive statistics were used for continuous data (mean ± standard deviation); categorical data were analyzed as frequencies or percentages and compared using the chi-square test. Participants were categorized according to whether they met homosexual partners at predetermined offline venues (1 = yes, 0 = no). Variables with P < 0.2 in the univariate regression analysis were included as independent variables in the multivariate logistic regression model using the Enter method. Statistical significance was set at P < 0.05.

## 3. Results

### 3.1 General demographic characteristics

A total of 604 participants were recruited in this study, with an average age of (28.04 ± 6.43) years. The youngest participant was 19 years old, while the oldest was 53 years old, with the age group of 26–53 years constituting 59.93% (362/604) of the sample. Individuals from rural areas accounted for 38.58% (233/604) of the participants. Approximately 41.39% (250/604) participantreported a monthly income exceeding 822 United States Dollars (USD). Those with college degrees or higher comprised 77.48% (468/604) of the participants. Students accounted for 20.53% (124/604) of the participants. Table 1 presents thedemographic characteristics of the participants

**Table 1:** Demographic characteristics of the participants.

Variables	Whether meeting homosexual partners at fixed offline locations						*x* ^*2*^	*P*
	Total (n = 604)		Yes (n = 133)		No (n = 471)			
	n	%	n	%	n	%		
**Age (yrs)**								
19–25	242	40.1	52	21.5	190	78.5	0.025	0.875
26–53	362	59.9	81	22.4	281	77.6		
**Residence registration**								
Urban areas	371	61.4	77	20.8	294	79.2	0.716	0.398
Rural areas	233	38.6	56	24.0	177	76.0		
**Educational level**								
High school or below	136	22.5	29	21.3	107	78.7	0.011	0.916
College degree or above	468	77.5	104	22.2	364	77.8		
**Student**								
No	480	79.5	106	22.1	374	77.9	<0.001	0.999
Yes	124	20.5	27	21.8	97	78.2		
Average monthly income (USD^*^)								
> 822	250	41.4	55	22.0	195	78.0	<0.001	1.00
≤ 822	354	58.6	78	22.0	276	78.0		

*USD, United states dollars.

### 3.2 Analysis of factors associated with meeting homosexualpartners at fixed offline locations

In this study, 133 participants met homosexual partners at fixed offline locations, such as bars, karaoke halls, and bathhouses, accounting for 22.02% (133/604) of the sample. Candidate variables identified through univariable screening (P < 0.2) encompassed: 1) whether they received HIV/AIDS prevention services in the past year, 2) willingness to engage in one-night stands, 3) openness to commercial sex, 4) recent anal intercourse with a homosexual partner, 5) seeking homosexual partners and engaging in casual sexual activities through the internet/dating apps in the past six months, 6) alcohol and drug use or use of erectile dysfunction medications (e.g., Viagra) during homosexual activities, 7) involvement in one-night stands with homosexual partners, 8) recent sexual activities with opposite-sex partners, 9) HIV testing in the past year, 10) use of PrEP in the last six months.

**Fig 1:**
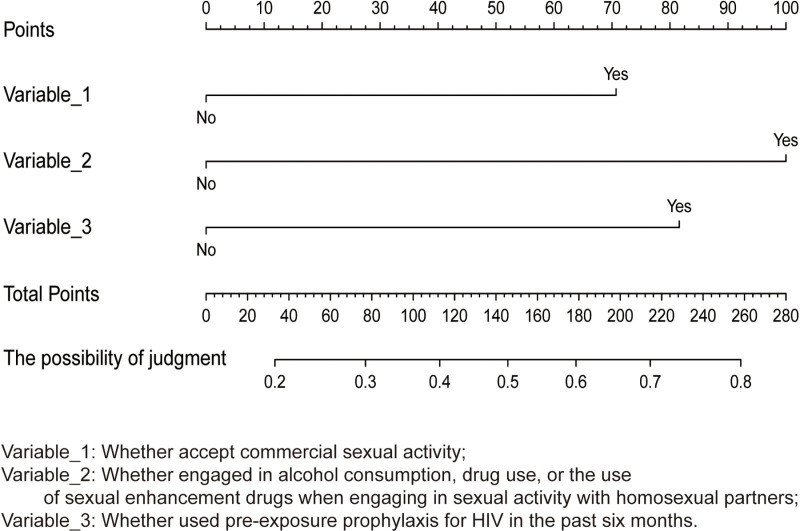
Nomogram of factors associatedwith participants’ meeting homosexual partners at fixed offline locations.

Variables with P < 0.2 in the univariate analysis were included in the multivariate logistic regression analysis, and the results (Table 2) indicated that participants willing to engage in commercial sex were more likely to meet homosexual partners at fixed offline locations (adjusted Odds Ratio [aOR]: 2.17; 95% Confidence Interval [CI]: 1.29–3.64); participants who reported alcohol consumption, drug use, or the use of erectile dysfunction medications during homosexual activities were more likely to meet homosexual partners at fixed offline locations (aOR: 3.06; 95% CI: 1.82–5.14); and participants who utilized PrEP in the last six months were more likely to meet homosexual partners at fixed offline locations (aOR: 2.17; 95% CI: 1.09–4.27).Nomogram of factors associatedwith participants’ meeting homosexual partners at fixed offline locations is shown in Fig 1.

**Table 2:** Analysis of factors associated with participants meeting homosexual partners at fixed offline locations.

Variables	Whether meeting homosexual partners at fixed offline locations				Univariate analysis		Multivariate analysis	
	Yes (n = 133)		No (n = 471)		*OR(95%CI)*	*P*	*aOR(95%CI)*	*P*
	n	%	n	%				
**Whether received HIV/AIDS prevention services in the past year**								
No	33	16.5	167	83.5	Ref.		Ref.	
Yes	100	24.8	304	75.2	1.66 (1.09-2.61)	0.022	1.51 (0.95-2.47)	0.087
**Whether accept one-night stands**								
No	52	16.1	271	83.9	Ref.		Ref.	
Yes	81	28.8	200	71.2	2.11 (1.43-3.14)	<0.001	1.05 (0.61-1.79)	0.861
**Whether accept commercial sexual activity**								
No	87	17.9	399	82.1	Ref.		Ref.	
Yes	46	39.0	72	61.0	2.93 (1.89-4.53)	<0.001	2.17 (1.29-3.64)	0.003
**Whether currently have a fixed homosexual partner**								
No	89	22.6	304	77.4	Ref.		——	——
Yes	44	20.9	167	79.1	0.90 (0.59-1.35)	0.612	——	——
**Whether have anal sex with the homosexual partner in the last six months**								
No	43	17.3	205	82.7	Ref.		Ref.	
Yes	90	25.3	266	74.7	1.61 (1.08-2.44)	0.021	1.05 (0.61-1.79)	0.863
**Whether consistently used condoms during anal intercourse with homosexual partners in the last six months**								
No	88	22.0	312	78.0	Ref.		——	——
Yes	45	22.1	159	77.9	1.003 (0.66-1.50)	0.987	——	——
**Whether meet homosexual partners and had casual sex through the Internet/dating software in the last six months**								
No	78	18.6	341	81.4	Ref.		Ref.	
Yes	55	29.7	130	70.3	1.85 (1.24-2.76)	0.003	1.13 (0.65-1.97)	0.66
**Whether engaged in alcohol consumption, drug use, or the use of sexual enhancement drugs when engaging in sexual activity with homosexual partners**								
No	93	18.0	425	82.0	Ref.		Ref.	
Yes	40	46.5	46	53.5	3.97 (2.46-6.42)	<0.001	3.06 (1.82-5.14)	<0.001
**Whether have ever engaged in one-night stands of homosexual activity**								
No	51	15.3	283	84.7	Ref.		Ref.	
Yes	82	30.4	188	69.6	2.42 (1.63-3.61)	<0.001	1.34 (0.80-2.25)	0.263
**Whether engaged in sexual activity with the heterosexual partner in the past six months**								
No	115	21.2	428	78.8	Ref.		Ref.	
Yes	18	29.5	43	70.5	1.56 (0.85-2.76)	0.139	1.11 (0.56-2.09)	0.758
**Whether had been tested for HIV in the past year**								
No	19	16.0	100	84.0	Ref.		Ref.	
Yes	114	23.5	371	76.5	1.62 (0.97-2.83)	0.077	1.13 (0.63-2.07)	0.694
**Whether used pre-exposure prophylaxis for HIV in the past six months**								
No	111	20.0	445	80.0	Ref.		Ref.	
Yes	22	45.8	26	54.2	3.39 (1.84-6.21)	<0.001	2.17 (1.09-4.27)	0.025

## 4. Discussion

The study findings revealed that 22% of participants encountered homosexual partners at specific offline venues. MSM constitute a high-risk demographic in the HIV epidemicthat is susceptible to HIV transmission owing to the unique nature of their sexual interactions [20]. MSMmay have increased partner turnover and sexual activity frequency, heightening the risk of HIV exposure [21]. Practices such asoral and anal sex facilitate direct mucosal entry of HIV, further increasing susceptibilityto infection [7,22]. MSM individuals often contend with discrimination, bias, and social exclusion in certain settings, presenting barriers to accessing health education and HIV testing services [12]. Insufficient knowledge of HIV, limited awareness of prevention methods, and inadequate understanding of sexual health can result in ineffective protective measures againstHIV infection [23]. Furthermore, epidemiological studies indicate that high-risk sexual behaviors prevalent among MSM populations, particularly concurrent multiple partnerships and inconsistent condom utilization, significantly elevate HIV transmission probabilities [24]. Promoting sexual health education, offering easily accessible health services (including HIV testing), reducing discrimination, and fostering social acceptance are vital strategies for mitigating the risk of HIV infection among MSM.

Commercial sex plays a significant role among MSM, potentially influencing the frequency and ways in which they meet homosexual partners [25,26]. Ourresults indicated that individuals open to commercial sex were more likely to encounter homosexual partners at designated offline venues, underscoring the social significance of commercial sex among MSM. Engaging in commercial sex may create opportunities for MSM to connect with homosexual partners and foster closer relationships through more frequent and intimate interactions that can lead to further sexual encounters [27]. Hence, individuals open to commercial sex are more predisposed to meeting homosexual partners through this avenue. Furthermore, commercial sex can provide a convenient and direct means of seeking sexual partners [28]. Transactional sexual interactions at fixed offline locations often serve as a means for MSM to establish social networks and intimate relationships, making it easier and faster to meet homosexual partners in such settings [29]. However, commercial sex is accompanied by risks such as the transmission of sexually transmitted infections, potential for violence, and mental health concerns [27]. Therefore, when examining the impact of commercial sex on how MSM engage with homosexual partners, it is vital to address these adverse aspects, implement appropriate prevention and intervention strategies, and safeguard their well-being [28,29]. These findings offer valuable insights into the role of commercial sex in MSM social interactions, and provide specific references for implementing sexual health education and preventive measures. To promote the health and safety of MSM, it is imperative to comprehensively consider the risk factors associated with commercial sex.

The co-occurrence of alcohol consumption, drug use, and aphrodisiac intake with sexual behavior may result in individuals experiencing impulsivity and impaired risk judgment during sexual encounters [30]. Under the influence of these substances, individuals may lower their partner selection standards, thereby increasing the likelihood of engaging with high-risk individuals [31]. Ourresults indicated that individuals who engage in alcohol consumption, drug use, or aphrodisiac intake during homosexual sexual encounters have a higher chance of meeting their sexual partners through offline venues. This finding underscores the significant role and impact of these risky behaviors on the social relationships of MSM and the importance of considering health risks within sexual relationships. Furthermore, offline venues serve as crucial hubs for social interaction among MSM, facilitating the formation of intimate relationships [32]. While these venues promote communication and information exchange for relationship building, substance use, such as alcohol, drugs, or aphrodisiacs, may escalate interactions, making participants more inclined towards engaging in sexual activities. However, these hazardous behaviors carry notable health risks, impacting both physical health and cognitive function, and can lead to impaired risk assessment and increased vulnerability to sexually transmitted diseases [33]. Consequently, it is crucial to raise awareness about the associated risks and implement appropriate interventions and support measures. A thorough examination of the correlation between these risky behaviors and sexual relationships is essential to bolster sexual health education and intervention initiatives, thereby mitigating the risk of sexually transmitted disease transmission and ensuring the well-being and safety of MSM during social interactions.

PrEP is widely recognized as an effective method for significantly reducing the risk of HIV transmission [34,35]. Individuals who have used PrEP are likely to show increased consideration of sexual health and HIV transmission risks when engaging with homosexual partners, leading them to adopt more vigilant and proactive measures to mitigate potential infection risks [5]. This proactive stance may drive them to prefer meeting their homosexual partners at offline venues, where they demonstrate heightened caution and awareness [36]. Our results indicatethat individuals who have taken PrEP in the preceding six months have higher probability of encountering homosexual partners at offline locations. This finding emphasizes the importance of preventive measures for sexual health. By integrating PrEP into health practices, individuals may develop a stronger sense of health responsibility, leading them to select partners who are conscious of risks and protective measures during sexual relationships [23]. This self-protective behavior encourages substantial conversations about safe sexual practices and health risk awareness with potential partners, favoring relationships with partners who prioritize health safety [36]. Despite the efficacy of PrEP in reducing HIV infection, understanding its scope, precautions, and potential side effects is crucial to avoid misuse and dependency [23,36]. While critical, HIV-prevention medication should not serve as a standalone alternative to other safe sexual practices such as condom use and risk reduction with casual partners. Comprehensive sexual health education and integrated health strategies are essential. In summary, PrEP influenced the pattern of encounters with homosexual partners among MSM, enhancing their preventive and self-protectionawareness [36]. These results advance our understanding of how preventive medication shapes sexual behavior, providing new insights and avenues for future research and practical applications in sexual health. Comprehensive preventive measures, education, advocacy, and holistic health management can enhance the sexual health status of MSM communities and reduce the risk of HIV infection.

Fixed offline locations offer a relatively safe and inclusive environment for social interactions among MSM [37]. These locations serve not only as spaces for seeking partners but also as opportunities for communication and trust-building, facilitating the development of deeper relationships [38]. For many MSM, offline venues are essential for establishing social networks, enabling them to explore sexual relationships in a more open and intimate manner [39]. Such individuals tend to be more attentive to sexual health and the risks associated with HIV transmission, often choosing to engage socially in these venues as a consideration for their well-being [38]. In these settings, individuals are also more likely to find partners who share similar health awareness, which is crucial for reducing the risk of HIV infection. However, it is important to note that socializing in these offline venues also presents certain risks, including sexually transmitted infections and potential violence. These risks may be exacerbated when alcohol, drugs, or aphrodisiacs are involved. Therefore, while encouraging MSM to form connections in fixed offline locations, it is equally vital to provide health education and risk prevention measures. Such initiatives can empower individuals to protect their health and that of their partners while pursuing social and sexual engagement. In summary, meeting in fixed offline locations significantly impacts the enhancement of social connections among MSM and the promotion of healthy behaviors. Consequently, further research on this phenomenon can deepen our understanding of its social significance and provide vital references for effective health intervention measures and educational strategies.

This study has several limitations. First, the study design is inherently constrained. As a cross-sectional study, it only offers data at a single research point anddoesnotshow the progression of events or causal connections. Future longitudinal cohort studies are required to effectively grasp the dynamic fluctuations and interrelations among variables. Second, the study sample lackedrepresentativeness and comprehensiveness. Due to the recruitment of MSM individuals via the internet, a potential selection bias could undermine the representativeness of the sample. Moreover, internet recruitment may fail to capture the entirety of the MSM population, thus compromising sample validity. To bolster the universality and applicability of the study findings, exploring alternative approaches, such as multichannel recruiting and collaborative efforts across multiple centers, may be beneficial. Additionally, deficiencies in data collection and analysis were apparent. Biases, data incompleteness, and potential errors in the self-reported information may have been present in this study. Furthermore, considering that MSM populations commonly encounter social discrimination and bias, concerns about privacy protection and societal acceptance may affect the participants’ engagement. Subsequent studies should address these aspects to enhance the generalizability of theconclusions.

## 5. Conclusions

Individuals who engage in commercial sex; have a history of alcohol consumption, drug use, or aphrodisiac use during homosexual encounters; and have utilized PrEP in the last six months are more prone to encountering homosexual partners at offline venues. It is advisable that during the development of strategies to curb HIV transmission, particular attention should be directed towards reinforcing interventions and awareness campaigns at fixed offline locations to mitigate high-risk sexual behaviors associated with MSM sexual practices. Further research can offer tailored intervention approaches for enhancing community health and preventing HIV infections, encompassing enhanced educational and awareness initiatives on commercial sex and the utilization of PrEP.

## Supporting information

S1 File Questionnaire. (DOC)
