# Supplementary material for: Factors associated with meeting homosexual partners at fixed offline locations among MSM recruited through the internet: A cross-sectional survey
Source: PLoS One. 2025 May 28;20(5):e0325273. doi: 10.1371/journal.pone.0325273 (PMC12118819; doi:10.1371/journal.pone.0325273)
Supplement: S1 File — (DOC) [file pone.0325273.s001.doc]

**Health Survey**

Hi, we are conducting a survey to understand people’s knowledge and behaviors regarding some health issues (such as HIV/AIDS or Monkeypox). This survey is anonymous, and we hope that your answers reflect your true personal situation. All information will be used for statistical analysis only, and your responses will be kept confidential. The survey will take approximately 10-15 minutes to complete. Your participation is important for our future prevention and control efforts. Thank you very much!

A01. Are you aware of the purpose and significance of this survey, knowledgeable about the content being surveyed, and do you agree to participate in this survey?
① Yes ② No

**If choose “yes” in A01, then continue answering the follow questions; otherwise, end the investigation.**

**Specific content of questionnaire survey:**

1. Year of your birth: ________ (Date format)
2. Marital status with the opposite sex: ① Single ② Married ③ Cohabiting ④ Divorced or Widowed
3. Registered residence: ① Urban ② Rural
4. Educational level: ① Primary school or below ② Junior high school or technical school ③ High school ④ Bachelor’s degree ⑤ Master’s degree or above
5. Occupation: ① Student ② Factory/Company Employee ③ Farmer ④ Migrant Worker ⑤ Administrative Staff ⑥ Teacher, Healthcare Worker ⑦ Entertainment Industry Worker ⑧ Other Service Industry Worker ⑨ Private Enterprise Owner ⑩ Other
6. Average monthly income in the past year (in CNY): ________ yuan
   ① Below 1000 ② 1001-3000 ③ 3001-6000 ④ Above 6001
7. Regarding your family: ① Parents have a good relationship ② Parents have an average relationship ③ Parents have a poor relationship ④ Parents are divorced
8. Which behavior is most likely to transmit HIV? ① Vaginal intercourse with a woman ② Anal intercourse with a woman ③ Anal intercourse with a man ④ Oral sex with a man ⑤ Don’t know
9. Which sexual role is more likely to contract HIV? ① Insertive partner ② Receptive partner ③ Both equally ④ Don’t know
10. Does using enhancers like Rush Popper or Zero Capsules increase the risk of HIV infection? ① Yes ② No ③ Don’t know
11. Can you visually tell if someone is infected with HIV/AIDS? ① Yes ② No ③ Don’t know
12. Can HIV be spread through mosquito bites? ① Yes ② No ③ Don’t know
13. Can you get HIV by having a meal with someone infected with HIV/AIDS? ① Yes ② No ③ Don’t know
14. Can you contract HIV by coming into contact with blood infected with HIV? ① Yes ② No ③ Don’t know
15. Is it possible to get HIV by sharing needles with someone infected with HIV? ① Possible ② Not possible ③ Don’t know
16. Can a child born to a woman with HIV/AIDS contract the virus? ① Possible ② Not possible ③ Don’t know
17. Does correct condom use reduce the transmission of HIV? ① Yes ② No ③ Don’t know
18. Does having sex with only one partner reduce the spread of HIV? ① Yes ② No ③ Don’t know
19. Have you recently obtained information about HIV/AIDS or Monkeypox through online media (Official accounts, QQ groups, apps, etc.)? ① Yes ② No
20. In the past year, have you received HIV/AIDS or Monkeypox prevention services (distribution of promotional materials and condoms, counseling services, training lectures, etc.)? ① Yes ② No
21. Are you open to one-night stands? ① Yes ② No ③ Don’t know
22. Are you open to commercial sex (involving monetary transactions like sponsorship, prostitution, selling sex)? ① Yes ② No ③ Don’t know
23. Are you open to homosexual activities of men who have sex with men (MSM)? ① Yes ② No ③ Don’t know
24. At what age did you join the MSM community?
25. What role do you play in homosexual activities of MSM? ① Insertive partner (1) ② Receptive partner (0) ③ Both (0.5)
26. How do you classify your sexual orientation? ① Homosexual ② Bisexual ③ Heterosexual ④ Undetermined
27. Do you currently have a stable homosexual partner? ① Yes ② No
28. At what age did you first engage in homosexual activities?
29. Have you met a homosexual partner through the internet (e.g., WeChat, QQ, Blued)? ① Yes ② No
30. At what age did you first engage in heterosexual activities (excluding homosexual activities)?
31. Have you met a homosexual partner through offline fixed locations (such as bars, KTVs, bathhouses)? ① Yes ② No
32. Where do you primarily find male sexual partners? ① Internet/Dating apps ② Tea rooms/Clubs ③ Bathhouses ④ Parks/Public restrooms/Fields ⑤ Bars/Nightclubs ⑥ Other (please specify)
33. In the last six months, have you engaged in anal sex with a homosexual partner? ① Yes ② No (Skip to question 40)
34. How many times have you engaged in anal sex with a homosexual partner in the last six months?
35. Did you use a condom the last time you engaged in anal sex with a homosexual partner in the last six months? ① Yes ② No
36. How often do you use condoms during anal sex with a homosexual partner in the last six months?
    ① Never ② Sometimes ③ Every time ④ Refuse to answer
37. In the last six months, have you sought and engaged in casual sexual activities with homosexual partners through the internet/dating apps? ① Yes ② No
38. How often, on average per month, do you seek and engage in casual sexual activities with homosexual partners through the internet?
    ① Never ② Occasionally (1%~20%) ③ Sometimes (21%~70%) ④ Frequently (71%~99%) ⑤ Every time
39. How often do you use condoms when engaging in casual sexual activities with homosexual partners through the internet?
    ① Never used ② Occasionally used (1%~20%) ③ Sometimes used (21%~70%) ④ Frequently used (71%~99%) ⑤ Always used
40. When engaging in sexual activities with a homosexual partner, have you engaged in the following behaviors? (Multiple choices possible)
    ① Drinking alcohol ② Drug use (including new drugs) ③ Use of enhancement drugs (e.g., Viagra) ④ None of the above
41. Have you engaged in the following homosexual behaviors? (Multiple choices possible) ① One-night stands ② Group sex ③ Sex parties ④ None of the above
42. In the last six months, have you engaged in commercial sexual activities with a homosexual partner? ① Yes ② No (Skip to question 45)
43. In the last six months, how frequently did you use condoms during commercial sexual activities with homosexual partners?
    ① Never used ② Occasionally used ③ Always used ④ Refuse to answer
44. In the last six months, did you use a condom the last time you engaged in commercial sexual activities with a homosexual partner? ① Yes ② No
45. In the last six months, have you engaged in sexual activities with heterosexual partners? ① Yes ② No (Skip to question 48)
46. In the last six months, how frequently did you use condoms during sexual activities with heterosexual partners?
    ① Never used ② Occasionally used ③ Always used ④ Refuse to answer
47. In the last six months, did you use a condom the last time you engaged in sexual activities with an heterosexual partner? ① Yes ② No
48. Have you ever used drugs? ① Yes ② No
49. In the past year, have you ever been diagnosed with a sexually transmitted infection (STI)? ① Yes ② No (Skip to question 51)
50. In the past year, which STIs have you been diagnosed with? (Multiple choices possible)
    ① Gonorrhea ② Syphilis ③ Chlamydia infection ④ Genital warts ⑤ Genital herpes ⑥ Other ⑦ None (No diagnosed STI in the past year)
51. Before engaging in sexual activities, do you feel confident discussing condom use with your sexual partner?
    ① Very confident ② Confident ③ Somewhat confident ④ Not confident ⑤ Not confident at all
52. If your sexual partner disagrees or does not have a condom during sexual activities, are you confident in abstaining from sex?
    ① Very confident ② Confident ③ Somewhat confident ④ Not confident ⑤ Not confident at all
53. Do you feel confident in purchasing condoms before sexual activities?
    ① Very confident ② Confident ③ Somewhat confident ④ Not confident ⑤ Not confident at all
54. Do you want to know the HIV status of your homosexual partners? ① Yes ② No
55. Do you know the HIV status of your homosexual partners? ① Yes ② No
56. Are you concerned about acquiring HIV through homosexual partners? ① Concerned ② Somewhat concerned ③ Not concerned
57. Do you believe that condoms can effectively prevent the transmission of HIV through homosexual partners? ① Very effective ② Ineffective or low effectiveness ③ Unsure
58. Have you ever been tested for HIV? ① Tested within the last year ② Tested a year ago ③ Never been tested (Skip to question 64)
59. How many times have you been tested for HIV? (If none, please fill in “0”)
60. When was your most recent test conducted? ______ year ______ month
61. Do you know the results of your most recent test? ① Yes ② No
62. How many self-rapid HIV tests have you taken in the past year? (If none, please fill in “0”)
63. What is your current HIV test result? ① Positive ② Negative ③ Unknown
64. What is the source of Monkeypox? (Multiple choices possible) ① Infected animals ② Infected humans ③ Uncertain
65. How does Monkeypox spread? (Multiple choices possible) ① Close contact with patients ② Respiratory droplets transmission ③ Contact with contaminated items ④ Vertical transmission ⑤ Sexual transmission ⑥ Uncertain
66. Groups susceptible to monkeypox include: (Multiple choices possible) ① Everyone ② Individuals not vaccinated for smallpox ③ Men who have sex with men ④ Individuals carrying HIV/AIDS ⑤ Uncertain
67. Clinical symptoms of monkeypox include: (Multiple choices possible) ① Chills and fever ② Swollen lymph nodes ③ Skin rashes on genital or perianal areas or other body parts ④ Weakness or fatigue ⑤ Muscle pain ⑥ Headache ⑦ Uncertain
68. Preventive measures for monkeypox include: (Multiple choices possible) ① Stay away from monkeypox outbreak areas ② Vaccination ③ Wearing masks ④ Hand hygiene ⑤ Having a regular partner and using condoms ⑥ Uncertain
69. Can taking specific medications at scheduled times reduce the risk of HIV infection before engaging in high-risk sexual activities? ① Yes ② No ③ Uncertain
70. Have you used pre-exposure prophylaxis for HIV within the last six months? ① Yes ② No
71. Can taking specific medications promptly reduce the risk of HIV infection after engaging in high-risk sexual activities? ① Yes ② No ③ Uncertain
72. Have you used post-exposure prophylaxis for HIV within the last six months? ① Yes ② No
